# A randomised placebo controlled trial of anakinra for treating pustular psoriasis: statistical analysis plan for stage two of the APRICOT trial

**DOI:** 10.1186/s13063-020-4103-z

**Published:** 2020-02-10

**Authors:** Suzie Cro, Prakash Patel, Jonathan Barker, David A. Burden, Christopher E. M. Griffiths, Helen J. Lachmann, Nick J. Reynolds, Richard B. Warren, Francesca Capon, Catherine Smith, Victoria Cornelius

**Affiliations:** 1 Imperial Clinical Trials Unit, Imperial College London, W12 7RH, London, UK; 2 St. John’s Institute of Dermatology, Guy’s and St Thomas’ NHS Foundation Trust, London, UK; 3 St. John’s Institute of Dermatology, School of Basic & Medical Biosciences, Faculty of Life Sciences & Medicine, King’s College London, London, UK; 4 Institute of Infection, Immunity and Inflammation, University of Glasgow, Glasgow, G12 8TA UK; 5 Centre for Dermatology Research, University of Manchester, NIHR Manchester Biomedical Research Centre, Manchester, UK; 6 National Amyloidosis Centre, University College London, NW3 2PF, London, UK; 7 Translational and Clinical Research Institute, University of Newcastle, Newcastle upon Tyne, NE1 7RU UK; 8 Dermatology Centre, Salford Royal NHS Foundation Trust, Manchester NIHR Biomedical Research Centre, The University of Manchester, Manchester, UK; 9 Department of Medical & Molecular Genetics, King’s College London, London, SE1 9RT UK

**Keywords:** Psoriasis, Palmoplantar pustulosis, Randomised controlled trial, Anakinra, Adaptive trial,, Statistical analysis plan

## Abstract

**Background:**

Current treatment options for Palmoplantar Pustulosis (PPP), a debilitating chronic skin disease which affects the hands and feet, are limited. The Anakinra for Pustular psoriasis: Response in a Controlled Trial (APRICOT) aims to determine the efficacy of anakinra in the treatment of PPP. This article describes the statistical analysis plan for the final analysis of this two-staged trial, which was determined prior to unblinding and database lock. This is an update to the published protocol and stage one analysis plan.

**Methods:**

APRICOT is a randomised, double-blind, placebo-controlled trial of anakinra versus placebo, with two stages and an adaptive element. Stage one compared treatment arms to ensure proof-of-concept and determined the primary outcome for stage two of the trial. The primary outcome was selected to be the change in Palmoplantar Pustulosis Psoriasis Area and Severity Index (PPPASI) at 8 weeks. Secondary outcomes include other investigator-assessed efficacy measures of disease severity, participant-reported measures of efficacy and safety measures. This manuscript describes in detail the outcomes, sample size, general analysis principles, the pre-specified statistical analysis plan for each of the outcomes, the handling of missing outcome data and the planned sensitivity and supplementary analyses for the second stage of the APRICOT trial.

**Discussion:**

This statistical analysis plan was developed in compliance with international trial guidelines and is published to increase transparency of the trial analysis. The results of the trial analysis will indicate whether anakinra has a role in the treatment of PPP.

**Trial registration:**

ISCRTN, ISCRTN13127147. Registered on 1 August 2016. EudraCT Number 2015-003600-23. Registered on 1 April 2016.

## Background

Palmoplantar pustulosis (PPP) is a debilitating chronic skin disease which affects the hands and feet. PPP produces intensely inflamed skin, covered with pustules. Unfortunately treatment options are currently limited [1]. Recent evidence suggests that interleukin-1 (IL-1), a cytokine known to sustain the inflammatory responses initiated by skin keratinocytes, may have a role in PPP [2–4]. Therefore, IL-1 blockade is hypothesized to be effective in the treatment of PPP.

Anakinra for Pustular Psoriasis: Response in a Controlled Trial (APRICOT) is a randomised, double-blind, placebo-controlled trial with two stages and an adaptive element which aims to determine the efficacy of anakinra in the treatment of adults with palmoplantar pustulosis (PPP). Full details on the rationale and background to the trial can be found in the published study protocol [5].

Since PPP is a rare condition and prior existing proof-of-concept data for anakinra are limited, APRICOT was designed to include two stages. Prior to the completion of a fully powered efficacy assessment (stage two), we compared the treatment arms at the end of stage one to provide reassurance for safety and evidence for potential treatment benefit. Stage one was also designed to confirm the primary outcome measure for the fully powered treatment comparison in stage two. The statistical analysis plan, which details the analyses undertaken in stage one, has previously been published [6]. The current document describes the statistical analyses to be undertaken at the end of stage two (the final analysis of the double-blind randomised controlled trial). The plan was approved by Catherine Smith (Chief investigator), Victoria Cornelius (Senior statistician), Suzie Cro (Trial Statistician) and Edel O’Toole (Trial Steering Committee chair) prior to database lock and to the unblinding of the trial statistician following database lock.

### Trial status

Recruitment to APRICOT began in October 2016. Stage one recruitment completed in September 2017 when a total of 24 patients had been randomised. Interim analysis at the end of Stage one, involving *n* = 24 patients, compared treatment arms to ensure sufficient efficacy following the pre-specified APRICOT stage one statistical analysis plan (SAP) [6]. The trial passed the stop/go efficacy criteria to progress to stage two, and a decision to embark on stage two, which involved a further 40 participants and was powered to determine efficacy, was made by the independent data monitoring committee (IDMC).

At the end of stage one, the IDMC assessed the distributions and reliability of two candidate outcomes to establish which one should be confirmed as the primary outcome. Following an assessment of reliability (as pre-specified in the stage one SAP [6]), the primary outcome for stage two was chosen to be the change in disease activity over 8 weeks, adjusted for baseline, measured using Palmoplantar Pustulosis Psoriasis Area and Severity Index (PPPASI).

In July 2019 an optional open label extension was added to the trial and offered to all patients who complete the 8-week treatment period and the 12-week follow up visit, including those who had completed the treatment period in previous years. Following slower than projected recruitment rates, all aspects of the trial design were critically reviewed, and an open label extension was one modifiable element. The primary purpose of the open label extension was to enhance the slow recruitment to the randomised, double-blind, placebo-controlled study, so that all participants have the potential opportunity to access anakinra. This change was informed by feedback from recruiting clinicians and patient reasons recorded for declining to take part in the trial. Recruitment to stage two is due to complete by the end of January 2020. Data collection for the double-blind randomised controlled trial is expected to complete by April 2020, and statistical analysis will be performed following data cleaning checks and database lock.

### Objectives

The primary objective of APRICOT is to determine the efficacy of anakinra on the change in disease activity over 8 weeks, measured using the PPPASI, in the treatment of adults with PPP compared to placebo.

#### Secondary objectives

The secondary objectives of the trial include the following:

- Estimate the efficacy of anakinra on the change in disease activity over 8 weeks, measured using pustule count compared to placebo
- Compare the time to response of PPP and the relapse rate with anakinra to placebo
- Estimate the proportion of patients who achieve clearance of PPP with anakinra compared to placebo by 8 weeks
- Estimate the treatment effect of anakinra in pustular psoriasis at non-acral sites
- Estimate the treatment effect of anakinra in plaque-type psoriasis
- Collate data on the adverse event profile and adverse reactions induced by anakinra and compare to placebo to evaluate the safety and tolerability of anakinra in the treatment of PPP
- Determine the effect of anakinra on patients quality of life compared to placebo
- Assess whether patients find treatment with anakinra acceptable or worthwhile
- Estimate adherence to treatment with anakinra

## Methods/Design

### Trial design

Anakinra for Pustular psoriasis: Response in a Controlled Trial (APRICOT) is a randomised, double-blind, placebo-controlled trial with two stages and an adaptive element, followed by an open label extension. Participants will be allocated to 8 weeks of treatment with either anakinra or placebo and will return for a visit 4 weeks after treatment is completed (12 weeks). All participants who complete the 8-week treatment period and the 12-week follow-up visit will be offered the open label extension on anakinra for 8 weeks. The protocol for the APRICOT trial has been published previously and gives full details on the intervention under study and the inclusion and exclusion criteria [5].

### Randomisation and blinding

Eligible participants with PPP will be randomised (1:1) to receive treatment with anakinra or placebo via subcutaneous daily injections for 8 weeks. To ensure allocation concealment, participants will be randomised using an online randomisation system by the King’s Clinical Trial Unit. Participants will be allocated to treatment arms using blocked randomisation stratified by centre.

Throughout the trial participants, research nurses, treating physicians and independent outcome assessors will be blinded to treatment assignment. The trial statistician will also be subgroup blind throughout the randomised trial. That is, the trial statistician will observe the data as group A versus group B, without knowing which treatments A and B refer to. The senior statistician who conducted the stage one analysis was subgroup-blind throughout stage one and was unblinded at the end of stage one.

### Outcomes

#### Primary outcome

The primary outcome is the disease activity at 8 weeks measured using the Palmoplantar Pustulosis Psoriasis Area and Severity Index (PPPASI), adjusted for baseline PPPASI.

#### Secondary outcomes

Secondary outcomes for APRICOT include the following:

Investigator assessed efficacy measures, which include

- Fresh pustule count on palms and soles at 8 weeks (measured at week 1, 4 and 8), adjusted for baseline
- Total pustule count on palms and soles at 8 weeks (measured at week 1, 4 and 8), adjusted for baseline
- PPP - Investigator’s Global assessment (PPP-IGA) at 8 weeks (measured at week 1, 4 and 8), adjusted for baseline
- Time from randomisation to response of PPP (where response is defined as a 75% reduction in fresh pustule count compared to baseline)
- Time from randomisation to relapse (where relapse is defined as return to baseline fresh pustule count)
- Achievement of ‘clear’ on PPP-IGA, at 8 weeks
- Development of a disease flare (i.e., > 50% deterioration in PPPASI compared to baseline) at 8 weeks
- Pustular psoriasis at non-acral sites (not hands and feet), as measured by percentage area of involvement at 8 weeks (measured at week 1, 4 and 8), adjusted for baseline
- Plaque type psoriasis (if present) measured using Psoriasis Area and Severity Index (PASI) at 8 weeks (measured at week 4 and 8), adjusted for baseline.

Participant reported efficacy outcomes, which include

- Participant’s Global Assessment (PGA, as clear, nearly clear, mild, moderate, severe, very severe) over 8 weeks adjusted for baseline (measured at 1, 4, and 8 weeks)
- Palmoplantar Quality of Life Instrument score at 8 weeks, adjusted for baseline
- Dermatology Life Quality Index (DLQI) at 8 weeks adjusted for baseline
- EQ5D-3 L at 8 weeks adjusted for baseline
- Treatment acceptability (i.e., whether the treatment is ‘worthwhile’) evaluated using a brief questionnaire with a response scale of 1–5 at week 12 (after the last treatment dose at the end of the study, prior to the final safety visit)
- Adherence to treatment measured by self-recall and responses to daily text messages over 8 weeks of treatment.

Safety measures, which include

- Serious infection as defined by any infection leading to death, hospital admission or requiring IV antibiotics
- Neutropenia (i.e., neutrophil count of ≤ 1.0 × 10^9^/l on at least one occasion)
- Serious adverse events (SAE), Serious adverse reactions (SAR), or Unexpected serious adverse reactions (USAR), which include any adverse event, adverse reaction or unexpected adverse reaction, respectively, that results in death, is life-threatening, required hospitalisation or prolongation of existing hospitalisation, results in persistent or significant disability or incapacity, or consists of a congenital anomaly or birth defect
- Adverse events (AE) and Adverse reactions (AR) including unexpected adverse reactions (UAR).

Additional exploratory/mechanistic outcomes will be collected during APRICOT. These outcomes will not contribute to the trial’s main findings and primary results publication, so they do not form part of the main trial statistical analysis plan and will be described in more detail in a separate analysis plan.

### Sample size

The sample size for APRICOT was undertaken at the design stage and prior to the completion of stage one, when the primary outcome of the main trial analysis was unknown. Consequently, the sample size was calculated using a standardised effect size. Given the high patient burden due to the requirement for daily self-administered subcutaneous injections and costs of the drug, an effect size of 0.9 Standard Deviations (SDs) was chosen to be the minimum important difference to detect with good power. Larger effect sizes have been reported when oral retinoids are used as the recommended systemic intervention for pustular psoriasis [1, 7]. With 90% power and a 5% significance level, for the detection of a difference of 0.9 SD, a sample size of 27 per arm is required. To allow for a conservative approximate 15% withdrawal rate, 32 participants per arm (*N* = 64 in total) are required.

After recruitment had been extended by an extra 12 months, by November 2019, 57 patients had been randomised into the APRICOT trial. The APRICOT Trial Management Group (TMG) met to discuss the lower than anticipated recruitment and the studies statistical power was considered for various sample size numbers as a contingency in the event of lower than planned recruitment. To detect a difference of 0.9 SD, with 80% power and a 5% significance level, while allowing for a conservative approximate 15% withdrawal rate, a sample size of 25 per arm (*N* = 50 in total) is required. Consequently, the decision was made to allow the trial to continue to recruit as many patients as possible within the funding constraints, given at least 80% power will be achieved.

### Statistical analysis plan

#### General analysis principles

The final (stage two) analysis will be performed after all recruited participants have completed 20 weeks of follow-up post-treatment initiation. Analyses will be carried out by the sub-group-blinded trial statistician and will follow the intention-to-treat (ITT) principle. That is, all eligible randomised participants with baseline and at least one recorded outcome (over 8 weeks) will be analysed in the treatment arms to which the participant was allocated, regardless of the treatment subsequently received. The safety set (SS) population consists of all participants who received at least one dose of the assigned intervention and will be used in the analysis to describe adverse events.

All regression analyses will include adjustment for centre where appropriate, as this was a stratification factor in the randomisation; therefore, inclusion of this adjustment is necessary in the analysis to maintain the correct type I error rate [8, 9]. Additionally, for continuous outcomes, the outcome measured at baseline will be included in regression analyses to increase the power [10]. All confidence intervals will be two-sided and at the 95% level. A *p*-value < 0.05 will be interpreted as statistically significant for the primary outcome.

#### Recruitment and participant flow

**Fig. 1:**
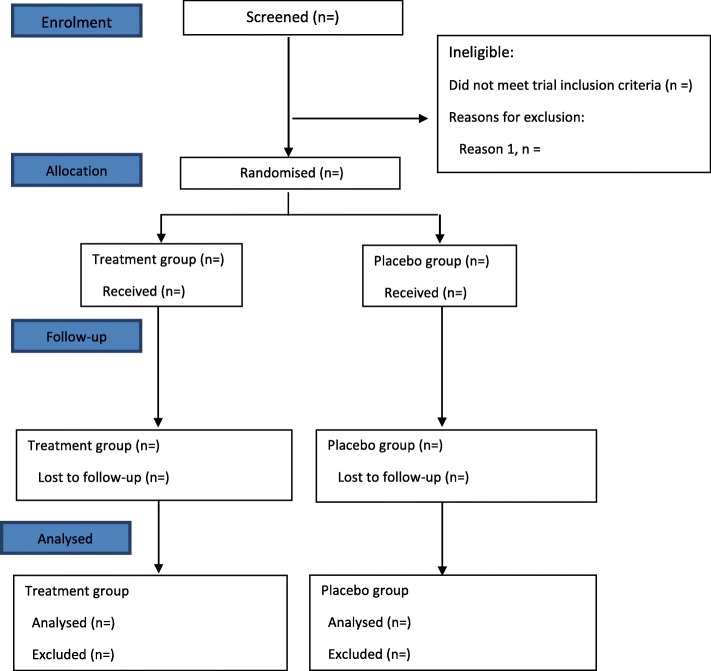
Template CONSORT diagram for APRICOT

The number of participants randomised will be summarised by treatment arm and study centre (Appendix in Table 3). To summarise the patient flow through the trial, a Consolidated Standards of Reporting Trials (CONSORT) flow chart will be constructed [11] *see* (Fig. 1). This will include the number of patients screened, eligible and randomised into the trial, withdrawing from treatment and lost to follow-up, and the number included in the analyses.

#### Baseline comparability of randomised groups

Baseline characteristics will be summarised by randomised treatment arm. The variables to be summarised are presented in Appendix in Table 4. Categorical variables will be summarised by number and percentage in each category. Continuous variables will be summarised by mean and standard deviation for approximately normally distributed variables or median and interquartile range for non-normally distributed variables. No formal statistical tests will be performed because any differences between treatment arms at baseline will be the result of chance rather than bias due to randomisation.

#### Withdrawals, loss to follow-up and missing data

The number withdrawing from the trial, including those lost to follow-up, will be reported by treatment arm and time point along with the reasons for the withdrawal. The overall loss to follow-up will be tabulated by treatment arm and visit. The proportions of participants missing PPPASI values (primary outcome) will be summarised in each arm and at each time point for which measurement is planned (see Appendix in Tables 5, 6, 7 and 8).

#### Adherence to allocated treatment

The number discontinuing the trial drug will be reported by treatment arm and week along with the reasons for the discontinuations (Appendix in Tables 9 and 10). Self-reported treatment adherence, as measured by responses to daily text messages and self-reported by patients using a paper trial diary or verbally self-recalled at study visits, will be reported by treatment arm and week for patients who have not yet discontinued the treatment or withdrawn from the study by the given week (Appendix in Table 11). An injection will be classed as being received if either a SMS response of ‘Yes’ is recorded for the day in question or if self-reported as a ‘Yes’. The adherence to the planned visit windows will also be summarised by treatment arm and visit (Appendix in Table 12).

#### Rescue therapy, topical therapy and prohibited medication

The proportion of participants using investigator-directed ‘rescue’ medication, as summarised in Table 1, in the form of potent corticosteroid (e.g., mometasone furoate, betamethasone valerate ointment or cream) and the duration of use and amount used will be summarised by treatment arm (Appendix in Tables 13, 14 and 15). We will plot histograms for the number of days of use of rescue therapy by treatment arm, plot the proportion of participants on rescue therapy over time and the cumulative proportion of participants initiated on rescue therapy over time.

**Table 1:** Summary of concomitant therapy rules

Prohibited	Very potent topical corticosteroids (e.g., Dermovate) Any topical treatment that is likely to impact signs and symptoms of PPP (e.g., corticosteroids, vitamin D analogues, calcineurin inhibitors, retinoids, keratolytics, tar or urea) Phototherapy or PUVA Methotrexate, Cyclosporine, Acitretin, Alitretinoin, FAE Etanercept or Adalimumab Infliximab or Ustekinumab or Secukinumab Other TNF antagonists Other systemic immunosuppressive therapy Other investigational monoclonal antibody Other investigational drugs
Allowable topical therapy	Emollients Topical hydrocortisone, antihistamine for injection – site reactions Mild topical corticosteroids for the treatment of psoriasis at sites other than hands and feet applied with gloves
‘Rescue’ topical therapy	Potent corticosteroid OD. To be dispensed only by the study team, at the Investigator’s discretion. Amounts prescribed to be recorded.

Where data allows, we will also summarise the overall proportion of participants using topical therapy during the treatment period, the duration of use and the amount used; histograms for the number of days of use of topical therapy will be constructed by treatment arm, and we will plot the proportion of participants on topical therapy over time.

If any prohibited medications are used (as defined in Table 1), we will also summarise the proportion of participants using prohibited medication, the prohibited medication used, the duration of use and the amount used.

#### Descriptive statistics for outcome measures

Descriptive statistics will be presented for all outcome measures by treatment arm. For each primary and secondary outcome that is recorded at multiple time points, the outcome will be summarised by visit and treatment arm (Appendix in Tables 16, 17, 18, 19, 20, 21, 22 and 23). Summary statistics with 95% confidence intervals will also be plotted in line graphs for each outcome across time by intervention. Only participants with a completely recorded outcome will be used to calculate the summary measures.

#### Adverse event reporting

Information on adverse events will be collected by means of spontaneous reports from participants and carers, clinical observation and clinical examinations and blood tests. A safety set (SS) population will be used for describing adverse events. This SS population will include all participants who receive at least one injection of study drug or placebo. For each event, local clinical investigators rate the relationship to the study medication as none/unlikely/possible/likely/definite. Adverse Reactions (AR) consist of the subset of non-serious adverse events (AE) rated to have a possible/likely/definite relationship with the study medication. Serious Adverse Reactions (SAR) consist of the subset of serious adverse events (SAE) rated to have a possible/likely/definite relationship with the study medication. If the event is considered related to the study medication, then local clinical investigators will also rate whether the reaction was unexpected (Yes/No). Events will be coded using terms of the clinical investigators choosing with reference to Medical Dictionary for Regulatory Activities (MedDRA) at the ‘Preferred Terms’ level.

Adverse events will be summarised by type (AE, AR, unexpected adverse reactions (UAR, a subset of the ARs), serious adverse events (SAE), serious adverse reactions (SAR, a subset of the SAEs) and unexpected serious adverse reactions (USAR, a subset of the SARs)), and by treatment arm. Adverse events will be tabulated by treatment group for both the number of events and the number of participants with the type of event.

A listing will be produced detailing all Serious Adverse Events (SAEs) and Reactions (SARs). Non-serious adverse events and reactions will be listed by MedDRA preferred term level. Non-serious adverse events will also be summarised by MedDRA system organ class and intensity (subjectively assessed by local clinical investigators as mild/moderate/severe). The number of events related to an infection will be tabulated. Details will be provided for those events related to infection, including the treatment prescribed, where applicable.

No hypothesis testing will be undertaken for adverse event outcomes, but approaches to assess signals for ARs will be explored, as described below (Appendix in Tables 24, 25, 26, 27, 28, 29, 30, 31 and 32). An adverse event of particular interest is injection site reaction. We will also separately analyse serious infections, defined by any infection leading to death, prolonged hospital admission or requiring IV antibiotics, as described below in further detail.

#### Analysis of primary outcome

The mean difference in the week 8 PPPASI, adjusted for baseline, between the two treatment groups will be estimated using a mixed-effects linear (Gaussian) regression model. The model will include a random intercept for participants with fixed effects for time, treatment group, time-by-treatment group interaction and baseline PPPASI. Centre will be included in the model either as a random or fixed effect depending on the total number of centres recruiting to the study and the average number of participants recruited from each centre. The estimated treatment effect at 8 weeks will be reported with a 95% confidence interval and corresponding *p*-value. As the model adjusts for baseline PPPASI, this is equivalent to analysing the change from baseline with adjustment for baseline [12]. The main conclusion of the trial will be based on this analysis time point. We will also report the treatment effect at weeks 1 and 4.

Approximately 15 centres are anticipated to recruit, and therefore, a relatively small number of participants will be recruited per centre. Since random centre effects have been shown to be superior to fixed effects in terms of power and precision when the number of participants per centre is small, and equivalent to fixed effects when the number of participants per centre is larger [9, 13], the default option will be to include centre as a random effect. With centre as a random effect, where *Y*_*ijk*_ denotes the PPPASI measurement for participant *i* at time *j* from centre *k*, the primary analysis model will be model A:

$$ {Y}_{ijk}={\beta}_0+{\beta}_1 TR{T}_i+{\beta}_2 PPPAS{I}_i^0+{\beta}_3{t}_4+{\beta}_4{t}_8+{\beta}_5{t}_4\ast TR{T}_i+{\beta}_6{t}_8\ast TR{T}_i+{b}_{1,i}+{b}_{2,k}+{e}_{ijk} $$

where j = 1 to 3 time points (week 1, 4, and 8), i = 1 to 64 participants, and k = 1 to ~ 15 centres; *TRT*_*i*_ is the dummy variable (*TRT*_*i*_ = 0 or 1) for participant *i*; ${PPPASI}_i^0$ is the baseline PPPASI for participant *i*; *t*_*x*_ is the dummy variable for time (= 0 or 1) at time point x weeks; Week 1 is represented by *t*_4_ = 0 and *t*_8_ = 0; and ${b}_{1,i}\sim N\left(0,{\sigma}_{b1}^2\right)$, ${b}_{2,k}\sim N\left(0,{\sigma}_{b2}^2\right)$, and ${e}_{ijk}\sim N\left(0,{\sigma}_e^2\right)$,

Within model A,*b*_1, *i*_ and *b*_2, *k*_ are random intercepts at the participant level and centre level, respectively. Each of *b*_1, *i*_, *b*_2, *k*_ and *e*_*ijk*_ are assumed to follow normal distributions. An unstructured covariance matrix will be used [14]. The treatment effect at 8 weeks, *β*_1_ + *β*_6_,will be of primary interest. If, however, the variation between centres is low and the model fails to converge, then centre will be treated as a fixed effect instead. With centre as fixed, where *y*_*ijk*_ denotes the PPPASI measurement for participant *i* at time *j* from centre *k*, the primary analysis model will be model B:

$$ {Y}_{ijk}={\beta}_0+{\beta}_1 TR{T}_i+{\beta}_2 PPPAS{I}_i^0+{\beta}_3{t}_4+{\beta}_4{t}_8+{\beta}_5{t}_4\ast TR{T}_i+{\beta}_6{t}_8\ast TR{T}_i+{\beta}_{7\mathrm{k}}{CENTRE}_i+{b}_{1,i}+{e}_{ijk} $$

where j = 1 to 3 time points (week 1, 4, and 8) and i = 1 to 64 participants; *TRT*_*i*_is the dummy variable (*TRT*_*i*_ = 0 or 1) for participant $i;{PPPASI}_i^0$ is the baseline PPPASI for participant *i*;

*t*_*x*_ is the dummy variable for time (= 0 or 1) at time point x weeks; Week 1 is represented by *t*_4_ = 0 and *t*_8_ = 0; *β*_7k_ is a dummy variable for each centre k; for centre 1, *β*_7k_ will be constrained to be 0; and${b}_{1,i}\sim N\left(0,{\sigma}_{b1}^2\right)$ and ${e}_{ijk}\sim N\left(0,{\sigma}_e^2\right)$,

Within model B, *b*_1, *i*_ is a random intercept at the participant level. Both *e*_*ijk*_ and *b*_1, *i*_ follow normal distributions. An unstructured covariance matrix will be used. The treatment effect at 8 weeks, *β*_1_ + *β*_6_,will be the mean treatment effect of primary interest.

Models will be fitted using restricted maximum likelihood (REML). Both models make assumptions about random effects distributions, correlation structure and residuals, which will all be investigated. If including centre as a fixed effect results in unstable model estimates, e.g., if a number of sites (> 1) have very few randomisations (≤3), we will exclude centre from the model (model C). Stata code for the primary outcome analysis is displayed in Table 2.

**Table 2:** Statistical analysis code for the primary outcome

Model	Analysis method	Stata code for analysis
A	Primary analysis: The PPPASI will be analysed using a linear mixed effects model, with fixed effects for time, treatment group, time-by-treatment group interaction and baseline PPPASI. The model will include a random intercept for participants and centre of recruitment.	mixed pppasi treat##i.time base_pppasi /// || centre: || id:, covariance(unstructured) reml
B	If Model A fails to converge: Model A without the random-effect for centre, centre will instead be included as a fixed effect	mixed pppasi treat##i.time base_pppasi i.centre /// || id:, covariance(unstructured) reml
C	If Model A fails to converge and > 1 site has ≤3 randomised: Primary analysis model excluding centre as a random or fixed effect	mixed pppasi treat##i.time base_pppasi /// || id:, covariance(unstructured) reml

#### Sensitivity analysis of primary outcome

Every effort will be made to obtain follow-up data for all participants including those who stop treatment. The primary analysis will include all observed data and employ maximum likelihood estimation.This approach is efficient for handling missing outcome data under the missing-at-random assumption (MAR). That is, the probability of missing data is assumed to not be dependent on the values of the unobserved data themselves, but instead conditional on the observed values of the variables included in the analysis model.

Sensitivity analyses addressing the impact of missing data will explore departures from the main MAR analysis assumption and potential missing not at random (MNAR) mechanisms using Multiple Imputation (MI) and a pattern mixture approach (Carpenter and Kenward, 2008) for all patients on the primary outcome following the ITT principle. Imputation under MAR will initially be performed separately within each treatment arm using chained equations following the guidance suggested by White et al. [15]. The variables in the imputation model will be the same as those in the analysis model without including more auxiliary variables after taking into account the relatively small sample size of this study [16]. Imputations will then be modified to investigate the impact of a better or poorer response than that predicted by MAR (lower/higher PPPASI scores) for participants with missing data. To do this, we will define δ as the postulated mean difference in the rate of change of the PPPASI score between the observed and unobserved cases for each week unobserved. For each participant in each intervention arm, we will then modify the MAR imputed observations accordingly by δ. Imputed data sets will be analysed using the primary analysis model. Results will be combined across imputed data sets using Rubin’s rules. We will repeat the analysis for a range of δ corresponding to +/− 10, 20, 30, 40 and 50% of the rate of change of the PPPASI over 8 weeks in all observed participants. We will also consider the possibility that data is missing informatively in one arm only. Only imputations for active arm participants will be modified for a range of δ corresponding to +/− 10, 20, 30, 40 and 50% of the rate of change of the PPPASI observed over 8 weeks in the active arm and the primary analysis repeated. Subsequently, only imputations for placebo participants will be modified as described above. Fifty imputations will be run for each MI analysis.

#### Supplementary analysis for the primary outcome

Four supplementary analyses are pre-planned for the trials primary outcome:

1. Supplementary analysis accounting for use of rescue therapy—Data after the initiation of rescue therapy will be set as missing. The primary analysis model will be fitted to data prior to the use of rescue therapy along with all observed data for patients who do not initiate rescue therapy to estimate the treatment effect in the absence of rescue therapy because participants who were initiated on rescue therapy are assumed to have had a similar outcome to those observed with the same history and profile in the absence of rescue therapy (MAR). Since the rescued participants would typically have had worse outcomes than those observed in the absence of rescue therapy, a pattern-mixture MI approach will subsequently be used to explore the impact of worse outcomes among the participants initiated on rescue therapy. Imputation under MAR will initially be performed as described above. We will define δ_R_ as the postulated mean difference in the rate of change of the PPPASI between the observed and rescued cases for each week post-rescue. For each participant initiating rescue therapy, we will then modify their MAR imputed observations accordingly by δ_R._ Imputed datasets will be analysed using the primary analysis model. Results will be combined across imputed datasets using Rubin’s rules. We will repeat the analysis for a range of δ_R_ corresponding to 10%, 20%, 30%, 40% and 50% of the change in the PPPASI observed in those with complete data over 8 weeks. For each MI analysis, 50 imputations will be run. These delta-adjusted sensitivity analyses will each provide an estimate of the treatment effect in the absence of rescue therapy, where participants who were initiated on rescue therapy are assumed to have had a specific poorer outcome than those observed with the same history and profile in the absence of rescue therapy.
2. Supplementary analysis accounting for use of prohibited medication—Data post initiation of rescue therapy and prohibited medication will be set as missing, and the analytical approach outlined above for accounting for rescue therapy will be adopted, but applied to individuals with data missing after rescue therapy or after prohibited medication use.
3. Supplementary analysis accounting for use of topical therapy—Where data allow, data will be set as missing when participants are on topical therapy at the time of the study follow-up visit. The primary analysis model will be fitted to data for participants not on topical therapy, along with all observed data for patients who do not initiate topical therapy. This analysis will provide an estimate of the treatment effect in the absence of topical therapy, under the assumption that participants who were initiated on topical therapy would have had a similar outcome to those observed with the same history and profile in the absence of topical therapy (MAR). Subsequently, data after the initiation of rescue therapy and prohibited medication will be set as missing, as well as data for participants while on topical therapy, and the analytical approach outlined above for accounting for rescue therapy will be adopted, but applied to individuals who are missing data from their time on topical treatment or after rescue therapy or prohibited medication use.
4. Supplementary analysis to estimate the complier average causal effect—For each participant, the proportion of injections received relative to injections planned (8 × 7 = 56) will be calculated and summarised based on the recorded daily adherence data. The complier average causal effect (CACE) will be estimated by a two-stage least squares instrumental variable regression for the primary endpoint (using ivregress 2sls in Stata). Here, we will initially define a ‘complier’ as an individual who complete more than 50% of the injections, that is, injections received relative to injections planned for the 8-week study period. Randomisation will be used as an instrumental variable for treatment received with adjustment for centre and baseline PPPASI on the week 8 outcome. Subsequently, we will alternatively define a ‘complier’ as an individual who completes ≥ 60%, ≥ 70%, ≥ 80% and ≥ 90% of injections, and we will employ the same analysis approach to address the impact of alternative definitions of compliance. If including centre as a fixed effect results in unstable model estimates, for example, if a number of sites have very few randomisations, we will exclude centre from the model and adjust for baseline PPPASI only.

#### Analysis of secondary outcomes

Continuous secondary outcomes (fresh pustule count, total pustule count, plaque-type psoriasis - PASI, palmoplantar quality of life instrument and EQ5D-3 L utility score) will each be analysed in a similar fashion to the primary PPPASI outcome using a linear mixed effects model. Similar to the primary analysis model, each model will include fixed effects for treatment group, time, treatment group-by-time interaction and baseline value of the associated outcome. A random patient intercept will also be included in each of the models. If convergence problems are experienced, the approach outlined for the primary outcome will be followed.

Binary secondary outcomes (clear on PPP-IGA, development of disease flare, serious infection, and neutropenia) will each be analysed with a mixed logistic regression model. The models will include a fixed effect for treatment group and centre as a random intercept. If convergence problems are experienced, the approach outlined for the primary outcome will be followed. The treatment odds ratio (OR) will be reported with 95% CI.

Ordinal secondary outcomes (PPP-IGA, PGA) will be analysed with mixed ordinal logistic regression models. The models will include a random intercept for participant and fixed effects for time, treatment group, time-by-treatment group interaction and baseline value of the outcome. Centre will be included as a random effect initially, but if non-convergence occurs, centre will be treated as a fixed effect or will be excluded if unstable model estimates occur. We will report the change in odds of a one-category increase in the outcome for patients in the active arm relative to placebo. The ordinal logistic regression model makes assumptions about proportional odds which will be checked for each outcome. If the proportional odds assumption appears to be strongly violated, a mixed effect multinomial logit model may alternatively be fitted.

For the time to event outcomes (time to response of PPP, time and to relapse), we will initially plot Kaplan Meier curves to visualise the unadjusted response rate over time by treatment group. Since outcomes are observed at relatively few discrete time intervals (week 1, 4, 8 and 12) random-intercept complementary log-log models will be used to estimate the treatment effects for the time to event outcomes. The model will include treatment group as a fixed effect and a random intercept for centre. The subject specific (conditional) hazard ratio for the treatment group will be reported with 95% CI. If convergence problems are experienced by including centre as a random effect, the approach outlined above will be followed. The complementary log-log model is appropriate for the discrete nature of the survival data [17]. The model corresponds to proportional hazards in continuous time. The proportional hazards assumption will be checked. If this assumption is violated, an alternative parameterisation will be used e.g., including a treatment-by-time interaction that varies the effect by time or restrictsing the observation time.

For adverse events and reactions, a volcano plot, constructed as described in [18], which plots the risk difference of the non-serious adverse events and reactions by the MedDRA preferred term between the treatment arms against the *p*-value from a Fishers’ exact test, will be examined to identify the events with the strongest evidence for between arm differences. A volcano plot will also be constructed to examine non-serious adverse events and reactions by MedDRA system organ class. Because few SAEs are anticipated, the SAEs will be evaluated individually, but they may also be included in these plots if thought to aid review. Where useful negative binomial or zero-inflated Poisson regression models will be used to estimate relative risks, risk differences and incidence rate ratios of non-serious events by MedDRA preferred term and/or system organ class. Where suitable, the timing of adverse events (using hazard plots) by treatment arm will be examined.

#### Exploratory analysis

A longitudinal analysis will be undertaken using a linear (Gaussian) mixed model to determine the treatment difference in PPPASI at 12 weeks. The analysis model will be the same as in the primary analysis but will include additional data at 12 weeks. The treatment effect for PPPASI at 12 weeks will be estimated and reported with 95% CI.

Since palmar disease may respond more quickly than plantar disease, exploratory analysis will separately estimate the efficacy of anakinra on the (i) disease activity at 8 weeks, measured using fresh pustule count on the palms, adjusted for baseline compared to placebo and (ii) disease activity at 8 weeks, measured using fresh pustule count on the soles, adjusted for baseline compared to placebo. For each of the palms and soles fresh pustule counts, a linear mixed effects model will be used, which includes fixed effects for treatment group, time, treatment group-by-time interaction, and baseline value of the associated outcome. A random patient intercept will also be included in each of the models. If convergence problems are experienced, the approach outlined for the primary outcome will be followed.

#### Missing baseline data

Missing baseline data are unlikely to be problematic for the analysis because the baseline values will be collected at the first clinic visit and centre will naturally be complete. However, if baseline values are missing, to avoid a loss of power within the analyses which adjust for baseline values, these values will be imputed with the mean baseline value calculated from the non-missing values using pooled data from both treatment groups. This technique improves the statistical efficiency in the estimation of the treatment effect and is justifiable since randomisation ensures that baseline values are independent of treatment group [19, 20].

#### Missing outcome data

The primary analysis will use all observed outcome data and will be conducted under the MAR assumption. As detailed above, we will conduct sensitivity analysis to assess the impact of departures from the MAR assumption on the results of the primary analysis. Secondary analyses will use all available outcome data and will also be conducted under the MAR assumption.

#### Interim analysis and data monitoring

The IDMC will review safety and efficacy data at time points of their choosing. No statistical hypothesis testing will be completed for the IDMC. Stage one analysis did not involve formal statistical hypothesis testing; as a result, no adjustment for interim analyses has been made.

#### Multiple comparisons

No multiplicity adjustments will be performed for the analysis of secondary outcomes, and results will be viewed as hypothesis-generating.

## Discussion

We have described in detail the planned analysis for the final stage (stage two) of APRICOT following the guidelines for the content of statistical analysis plans in clinical trials [21]. The APRICOT trial will establish the role of anakinra in treating PPP. This pre-specified statistical analysis plan will increase the transparency of the data analysis and reporting.

### Trial registration

ISCRTN ISCRTN13127147 registered on 1 August 2016, http://www.isrctn.com/ISRCTN13127147. EudraCT Number: 2015–003600-23 registered on 1 April 2016, https://www.clinicaltrialsregister.eu/ctr-search/search?query=2015-003600-23.

## Data Availability

The protocol and statistical analysis plan can be obtained by contacting the corresponding author. The study team will retain the exclusive use of data until publication of major outputs has been completed, when data may be obtained from the chief investigator upon reasonable request.

## Appendix

**Table 3:** Randomisation by centre and treatment group

	Group A *N* (%)	Group B *N* (%)	Total *N*
Number randomisation to date (dd/mm/yyyy)			
Study centre			
Centre 1			
Centre 2			
Centre 3			
Centre 4			

**Table 4:** Baseline demographics by treatment group

Characteristic	Group A N=	Group B N=	Total N=
Age			
N (Nmissing)			
Mean (SD)			
Median (IQR)			
Min-Max			
Gender			
N (Nmissing)			
Male, n (%)			
Female, n (%)			
Ethnicity			
White, n (%)			
Asian or Asian British, n (%)			
Black or Black British, n (%)			
Chinese, Japanese, Korean, Indochinese, n (%)			
Mixed race, n (%)			
Other, n (%)			
Smoking status			
Current smoker, n (%)			
Ex-smoker, n (%)			
Non-smoker, n (%)			
PPPASI			
N (Nmissing)			
Mean (SD)			
Median (IQR)			
Min-Max			
Fresh pustule count (palms and soles)			
N (Nmissing)			
Mean (SD)			
Median (IQR)			
Min-Max			
Fresh pustule count (palms)			
N (Nmissing)			
Mean (SD)			
Median (IQR)			
Min-Max			
Fresh pustule count (soles)			
N (Nmissing)			
Mean (SD)			
Median (IQR)			
Min-Max			
Total pustule count (palms and soles)			
N (Nmissing)			
Mean (SD)			
Median (IQR)			
Min-Max			
PPP-IGA			
N (Nmissing)			
Clear			
Almost clear			
Mild			
Moderate			
Severe			
Participant global assessment			
N (Nmissing)			
Clear			
Almost clear			
Mild			
Moderate			
Severe			
Very severe			
DLQI			
N (Nmissing)			
Mean (SD)			
Median (IQR)			
Min-Max			
Pustular psoriasis at non acral sites (BSA)			
N (Nmissing)			
Mean (SD)			
Median (IQR)			
Min-Max			
Generalised plaque psoriasis – PASI			
N (Nmissing)			
Mean (SD)			
Median (IQR)			
Min-Max			
Palmoplantar quality of life instrument			
N (Nmissing)			
Mean (SD)			
Median (IQR)			
Min-Max			
EQ 5D- utility score			
N (Nmissing)			
Mean (SD)			
Median (IQR)			
Min-Max			
EQ 5D-VAS			
N (Nmissing)			
Mean (SD)			
Median (IQR)			
Min-Max			

**Table 5:** Withdrawals from trial by treatment group

Point of Withdrawal:	Group A N=	Group B N=	Total N=
Baseline date (n, %)			
By week 1 (n, %)			
By week 4 (n, %)			
By week 8 (n, %)			
By week 12 (n, %)			
By week 20 (n, %)			

**Table 6:** Reasons for withdrawal by treatment group

Reason	Group A N=	Group B N=	Total N=
Lost to follow-up (n, %)			
Reason 2 (n, %)			
Reason 3 (n, %)			
Reason 4 (n, %)			

**Table 7:** Loss to follow-up

Loss to follow-up	Group A N =	Group B N =	Total N =
Baseline			
Week 1 (*n*, %)			
Week 4 (*n*, %)			
Week 8 (*n*, %)			
Week 12 (n, %)			

**Table 8:** Missing data for PPPASI

Missing	Group A *N* =	Group B *N* =	Total *N* =
Baseline			
Week 1 (*n*, %)			
Week 4 (*n*, %)			
Week 8 (*n*, %)			
Week 12 (*n*, %)			

**Table 9:** Permanent trial drug discontinuation

Point of treatment discontinuation	Group A *N*=	Group B *N*=	Total *N*=
Baseline (*n*, %)			
Week 1 (*n*, %)			
Week 2 (*n*, %)			
Week 3 (*n*, %)			
Week 4 (*n*, %)			
Week 5 (*n*, %)			
Week 6 (*n*, %)			
Week 7 (*n*, %)			
Week 8 (*n*, %)			

**Table 10:** Reasons for trial drug discontinuation

Reason	Group A *N*=	Group B *N*=	Total *N*=
Reason 1 (*n*, %)			
Reason 2 (*n*, %)			
Reason 3 (*n*, %)			
Reason 4 (*n*, %)			

**Table 11:** Self-reported adherence to treatment group

Treatment period	Mean number of doses per week (SMS)			Mean number of doses per week (Diary)			Mean number of doses per week (Total*)		
	N	Group A Mean (SD)	Group B Mean (SD)	N	Group A Mean (SD)	Group B Mean (SD)	N	Group A Mean (SD)	Group B Mean (SD)
Week 1									
Week 2									
Week 3									
Week 4									
Week 5									
Week 6									
Week 7									
Week 8									

*SMS and Diary data combined

**Table 12:** Adherence to visit windows

Visit	Difference |expected – actual|			
	Group A *N*=		Group B *N*=	
	*N*	Mean (min, max)	*N*	Mean (min, max)
Baseline				
Week 1				
Week 4				
Week 8				
Week 12				
Week 20				

**Table 13:** Rescue therapy by treatment group

Treatment given	Group A *N*=	Group B *N*=	Total *N*=
Treatment 1 (*n*, %)			
Treatment 2 (*n*, %)			
Treatment 3 (*n*, %)			
Total			

**Table 14:** Rescue therapy by treatment group and time point of first initiation

Rescue initiation	Group A *N*=	Group B *N*=	Total *N*=
Prior to week 1 (*n*)			
Prior to week 4 (*n*)			
Prior to week 8 (*n*)			
Prior to week 12 (*n*)			
Prior to week 20 (*n*)			
Total			

**Table 15:** Days of use of rescue therapy and amount used

Rescue therapy	Treatment group	
	A	B
Number of patients taking rescue therapy		
Total no. of days of use		
Mean, SD		
Median, IQR		
Min, Max		
Total amount used (g)		
Mean, SD		
Median, IQR		
Min, Max		
Average amount per use (g)		
Mean, SD		
Median, IQR		
Min, Max		

**Table 16:** Primary outcome, PPPASI

Time	Treatment Group						Total *N*	Unadjusted Mean Difference: (95% CI)
	A			B				
	*N*	Mean	SD	*N*	Mean	SD		
Baseline								
Week 1								
Week 4								
Week 8								
Week 12								

**Table 17:** Fresh pustule count

Time	Treatment Group						Total *N*	Unadjusted Mean Difference: (95% CI)
	A			B				
	*N*	Mean	SD	*N*	Mean	SD		
Baseline								
Week 1								
Week 4								
Week 8								
Week 12								

**Table 18:** Total pustule count

Time	Treatment Group						Total *N*	Unadjusted Mean Difference: (95% CI)
	A			B				
	*N*	Mean	SD	*N*	Mean	SD		
Baseline								
Week 1								
Week 4								
Week 8								
Week 12								

**Table 19:** PPP-IGA

Time	*N*					
		Clear	Almost clear	Mild	Moderate	Severe
Group A						
Baseline						
Week 1						
Week 4						
Week 8						
Week 12						
Group B						
Baseline						
Week 1						
Week 4						
Week 8						
Week 12						

**Table 20:** Pustular psoriasis at non-acral sites (body surface area)

Time	Treatment Group						Total *N*	Unadjusted Mean Difference: (95% CI)
	A			B				
	*N*	Mean	SD	*N*	Mean	SD		
Baseline								
Week 1								
Week 4								
Week 8								
Week 12								

**Table 21:** Plaque type psoriasis (PASI)

Time	Treatment Group						Total *N*	Unadjusted Mean Difference: (95% CI)
	A			B				
	*N*	Mean	SD	*N*	Mean	SD		
Baseline								
Week 4								
Week 8								
Week 12								

**Table 22:** Participants global assessment

Time	*N*	PGA – *N* (%)					
		Clear	Nearly clear	Mild	Moderate	Severe	Very severe
Group A							
Baseline							
Week 1							
Week 4							
Week 8							
Week 12							
Group B							
Baseline							
Week 1							
Week 4							
Week 8							
Week 12							

**Table 23:** Palmoplantar quality of life, DLQI and EQ. 5D-3 L Index

Outcome	Time	Treatment group						Total *N*	Unadjusted mean difference: (95% CI)
		A			B				
		*N*	Mean	SD	*N*	Mean	SD		
Palmoplantar QoL	Baseline								
	Week 8								
DLQI	Baseline								
	Week 8								
EQ 5D-3 L utility	Baseline								
	Week 8								

**Table 24:** Summary of safety events type by treatment group

Event type	Number of Events			Number of Participants with Events		
	Group A *N* =	Group B *N* =	Total *N* =	Group A *N* =	Group B *N* =	Total *N* =
All events						
AE/AR						
UAR						
SAE/SAR						
USAR						

**Table 25:** Serious adverse events and reactions by treatment group

Id	Serious adverse reaction	Group	Onset date	Resolved date	Intensity	Relatedness
Id	Serious adverse event	Group	Onset date	Resolved date	Intensity	Relatedness

**Table 26:** Adverse events and reactions by body system class and treatment group

Body system code	Number of events			Number of participants with events		
	Group A *N* =	Group B *N* =	Total *N* =	Group A *N* =	Group B *N* =	Total *N* =
Respiratory						
Gastro-intestinal						
Genito-urinary						
Musculo-skeletal						
Neurological						
Immunological						
Dermatological						
Allergies						
Eyes, ear, nose, throat						
Other						
Missing						

**Table 27:** Adverse events at preferred term level by treatment group

AE term	Group A *N* events	Group B *N* events	Total *N* events
Mild events			
Moderate events			
Severe events			

**Table 28:** Adverse reactions at preferred term level by treatment group

AE term	Group A *N* events	Group B *N* events	Total *N* events
Mild events			
Moderate events			
Severe events			

**Table 29:** Summary of harm events by relatedness to infection, event type and treatment group

Event Type	Total number of events		Related to an infection				Not related to an infection			
			Number of events		Number of participants		Number of events		Number of participants	
	A	B	A	B	A	B	A	B	A	B
Adverse event										
Adverse reaction										
Unclassified (non-serious)										
Serious adverse event										
Serious adverse reaction										
Total										

**Table 30:** Numbers prescribed medication for harm events related to an infection by event type and treatment group

Event type	Total number of events related to infection		Prescribed Medication			
			Number of events		Number of participants	
	A	B	A	B	A	B
Adverse event						
Adverse reaction						
Unclassified (non-serious)						
Serious adverse event						
Serious adverse reaction						
Total						

**Table 31:** Details of adverse events which relate to an infection by treatment group

id	Event term*	Start date	On-going	Stop date	Intensity	Related to IMP	Medication prescribed	Details of medication prescribed
Group A								
Group B								

* Indicates study medication was temporarily interrupted

**Table 32:** Details of adverse reactions which relate to an infection by treatment group

id	Event term*	Start date	On-going	Stop date	Intensity	Related to IMP	Medication prescribed	Details of medication prescribed
Group A								
Group B								

* Indicates study medication was temporarily interrupted

## Abbreviations

AE: adverse event
APRICOT: Anakinra for Pustular Psoriasis: Response in a Controlled Trial
AR: adverse reaction
CACE: complier average causal effect
CI: confidence interval
CONSORT: Consolidated Standards of Reporting Trials
DLQI: Dermatology Life Quality Index
EME: Efficacy and Mechanism Evaluation Programme
IDMC: independent data monitoring committee
IGA: Investigators Global Assessment
ITT: intention-to-treat
MAR: missing at random
MedDRA: Medical Dictionary for Regulatory Activities
MI: multiple imputation
MNAR: missing-not-at-random
PASI: Psoriasis Area and Severity Index
PGA: Participant’s Global Assessment
PPP: palmoplantar pustulosis
PPPASI: Palmoplantar Pustulosis Area and Severity Index
PPP-IGA: Palmoplantar Pustulosis Investigators Global Assessment
REML: restricted maximum likelihood
SAE: serious adverse event
SAR: serious adverse reaction
SD: standard deviation
SOBI: Swedish Orphan Biovitrum
SS: safety set
UAR: unexpected adverse reaction
USAR: unexpected serious adverse reaction
